# Quantitative assessment and objective improvement of the accuracy of neurosurgical planning through digital patient-specific 3D models

**DOI:** 10.3389/fsurg.2024.1386091

**Published:** 2024-04-24

**Authors:** Sahin Hanalioglu, Muhammet Enes Gurses, Baylar Baylarov, Osman Tunc, Ilkay Isikay, Nergiz Ercil Cagiltay, Ilkan Tatar, Mustafa Berker

**Affiliations:** ^1^ Department of Neurosurgery, Faculty of Medicine, Hacettepe University, Ankara, Turkey; ^2^ BTech Innovation, METU Technopark, Ankara, Turkey; ^3^ Department of Software Engineering, Cankaya University, Ankara, Turkey; ^4^ Department of Anatomy, Faculty of Medicine, Hacettepe University, Ankara, Turkey

**Keywords:** 3D model, surgical planning, simulation, assessment, education, brain tumor

## Abstract

**Objective:**

Neurosurgical patient-specific 3D models have been shown to facilitate learning, enhance planning skills and improve surgical results. However, there is limited data on the objective validation of these models. Here, we aim to investigate their potential for improving the accuracy of surgical planning process of the neurosurgery residents and their usage as a surgical planning skill assessment tool.

**Methods:**

A patient-specific 3D digital model of parasagittal meningioma case was constructed. Participants were invited to plan the incision and craniotomy first after the conventional planning session with MRI, and then with 3D model. A feedback survey was performed at the end of the session. Quantitative metrics were used to assess the performance of the participants in a double-blind fashion.

**Results:**

A total of 38 neurosurgical residents and interns participated in this study. For estimated tumor projection on scalp, percent tumor coverage increased (66.4 ± 26.2%–77.2 ± 17.4%, *p* = 0.026), excess coverage decreased (2,232 ± 1,322 mm^2^–1,662 ± 956 mm^2^, *p* = 0.019); and craniotomy margin deviation from acceptable the standard was reduced (57.3 ± 24.0 mm–47.2 ± 19.8 mm, *p* = 0.024) after training with 3D model. For linear skin incision, deviation from tumor epicenter significantly reduced from 16.3 ± 9.6 mm–8.3 ± 7.9 mm after training with 3D model only in residents (*p* = 0.02). The participants scored realism, performance, usefulness, and practicality of the digital 3D models very highly.

**Conclusion:**

This study provides evidence that patient-specific digital 3D models can be used as educational materials to objectively improve the surgical planning accuracy of neurosurgical residents and to quantitatively assess their surgical planning skills through various surgical scenarios.

## Introduction

Neurosurgical training requires profound anatomical knowledge complemented with acquisition of visuospatial and fine motor skills (1–3). One of the main challenges in neurosurgical training is to comprehend the complex 3D relationships of pathological and anatomical structures, and apply this knowledge to surgical operations (3, 4). This requires dedication, effort, and practice as well as the use of various educational resources and tools to maximize learning experiences (1, 3, 5–10).

A wide spectrum of educational tools from cadaver dissections to virtual 3D models and simulators have been widely used to enhance training of neurosurgeons (11–13). Although generic models are useful to learn anatomy and normal or pathological variations, their potential to complement surgical planning skills for specific cases are limited since they do not reflect exact pathological anatomy of individual patients.

With the advent of neuroimaging and image processing technologies, patient-specific 3D models, either digital or printed, can now be produced and used for surgical planning simulations (14–17). Neurosurgical patient-specific 3D printed models have been shown to facilitate learning, enhance planning skills and improve surgical results in brain tumor, skull base and cerebrovascular surgeries (14–19). However, they have certain disadvantages such as need for special equipment and materials, higher costs, lengthy production process, lack of repetitive use, which limit their utility in every-day practice and training of neurosurgical residents (14, 18, 20, 21). On the other hand, high quality, multilayered digital 3D images or models can be easily produced with limited resources and adopted in the routine neurosurgical planning pipeline. Despite several studies published in recent years (22, 23), literature is scarce for the validation of these personalized-3D virtual models as both educational and assessment tools.

## Materials and methods

### Study design

**Figure 1:**
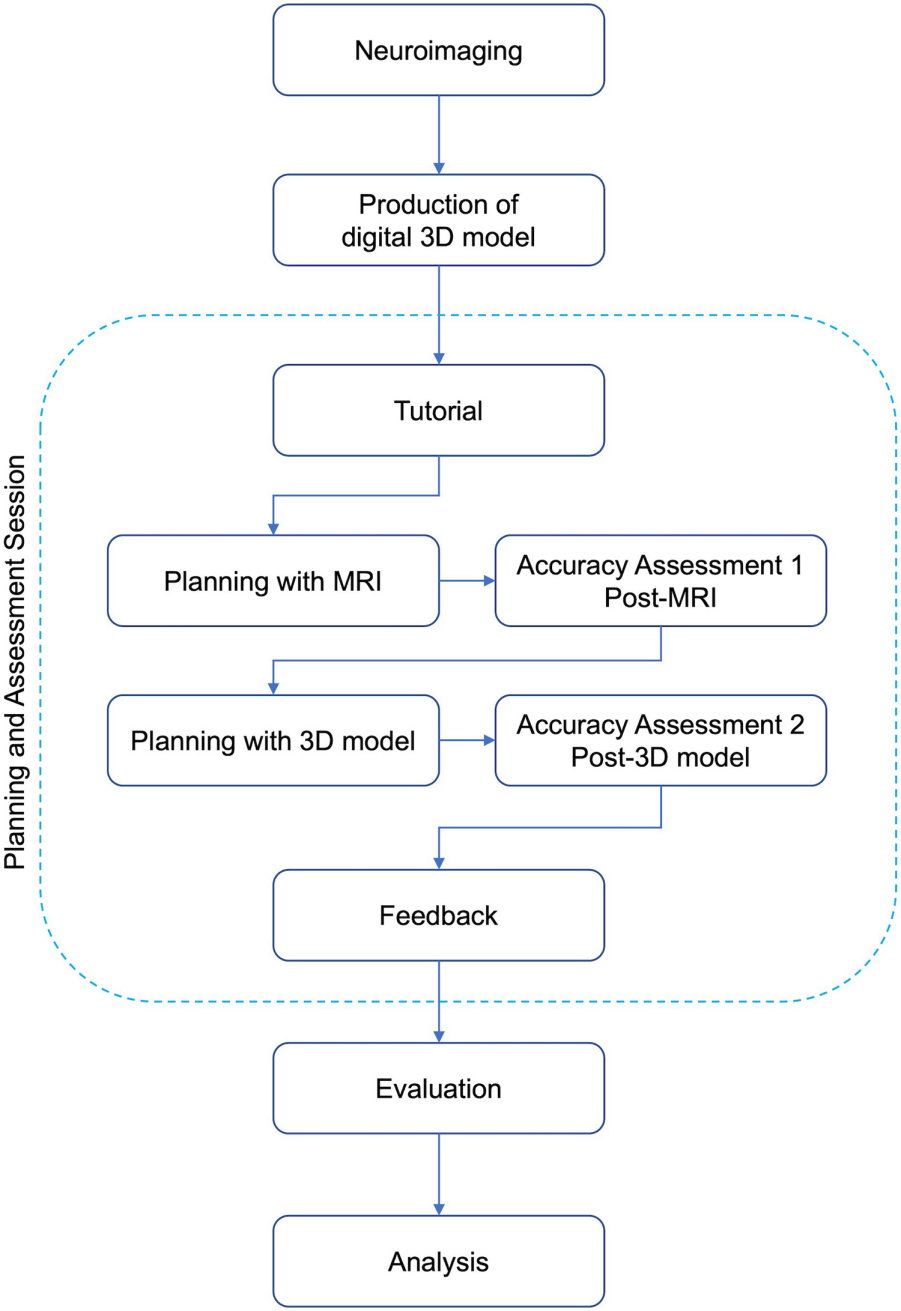
Study flowchart.

This study was conducted as a proof-of-concept study on current neurosurgical residents and interns (who completed one-month clinical rotation in neurosurgery) in one university hospital. The study was designed to demonstrate whether adding a brief practice with patient-specific digital 3D model to conventional surgical planning session with 2D images (axial, coronal and sagittal MRI) has any effect on the accuracy of surgical planning process of the residents. The design of the study is illustrated as a diagram in Figure 1. Briefly, the study included three phases: 1) preparation, (2) planning and assessment session, (3) evaluation and analysis.

For the preparation phase, a brain tumor case was selected via search of our institutional database with the following criteria: (i) common pathology, (ii) good quality neuroimaging available, (iii) proximity to critical neurovascular structures, (iv) requirement for a relatively standard surgical planning.

Planning and assessment were performed for each neurosurgical resident separately in a face-to-face session using a structured methodology described below. Lastly, assessment of the performances was retrospectively conducted by using digital data collected during the accuracy assessment session.

### Reconstruction of patient-specific 3D model

**Figure 2:**
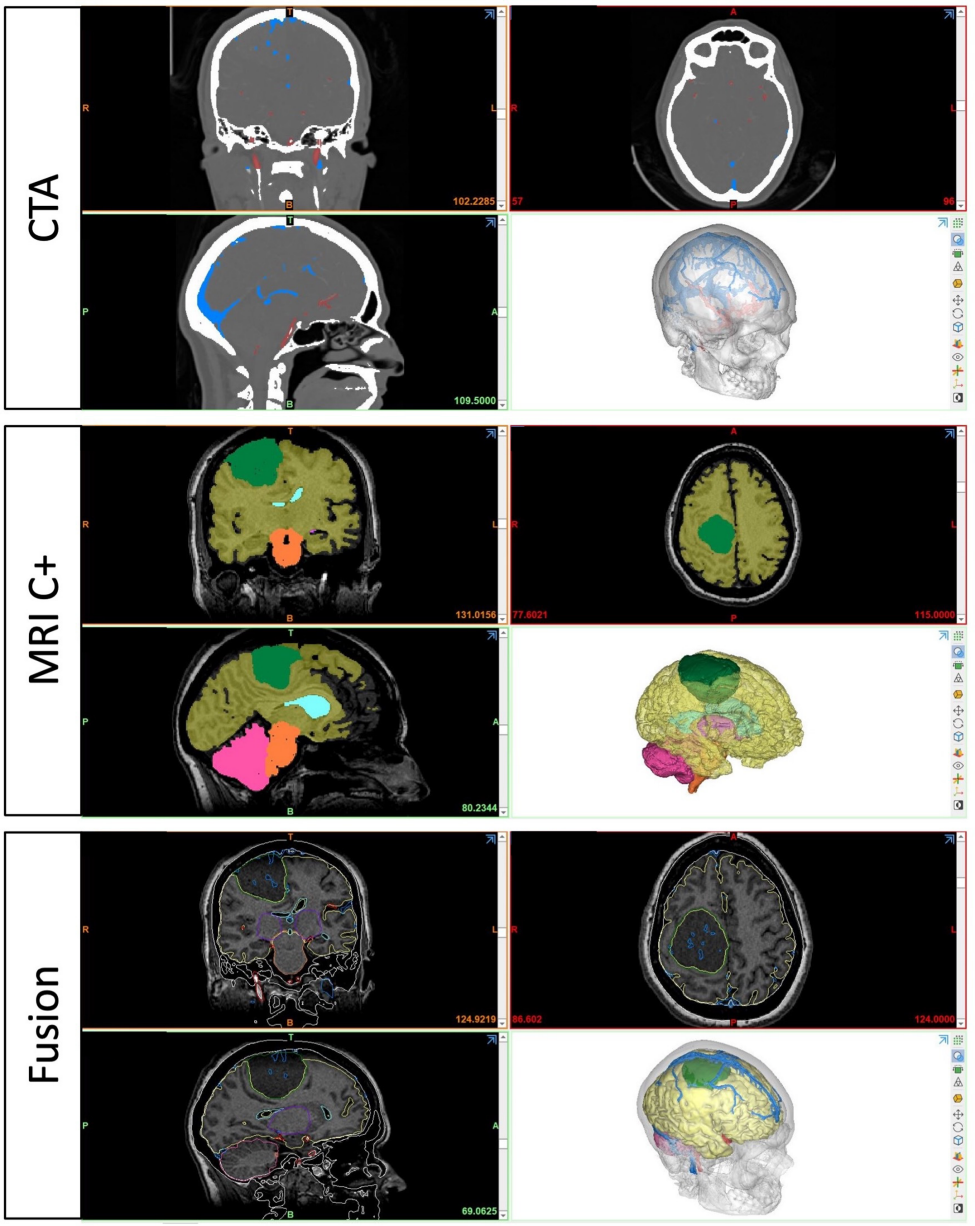
3-dimensional rendering and segmentation process using different neuroimaging modalities.

**Figure 3:**
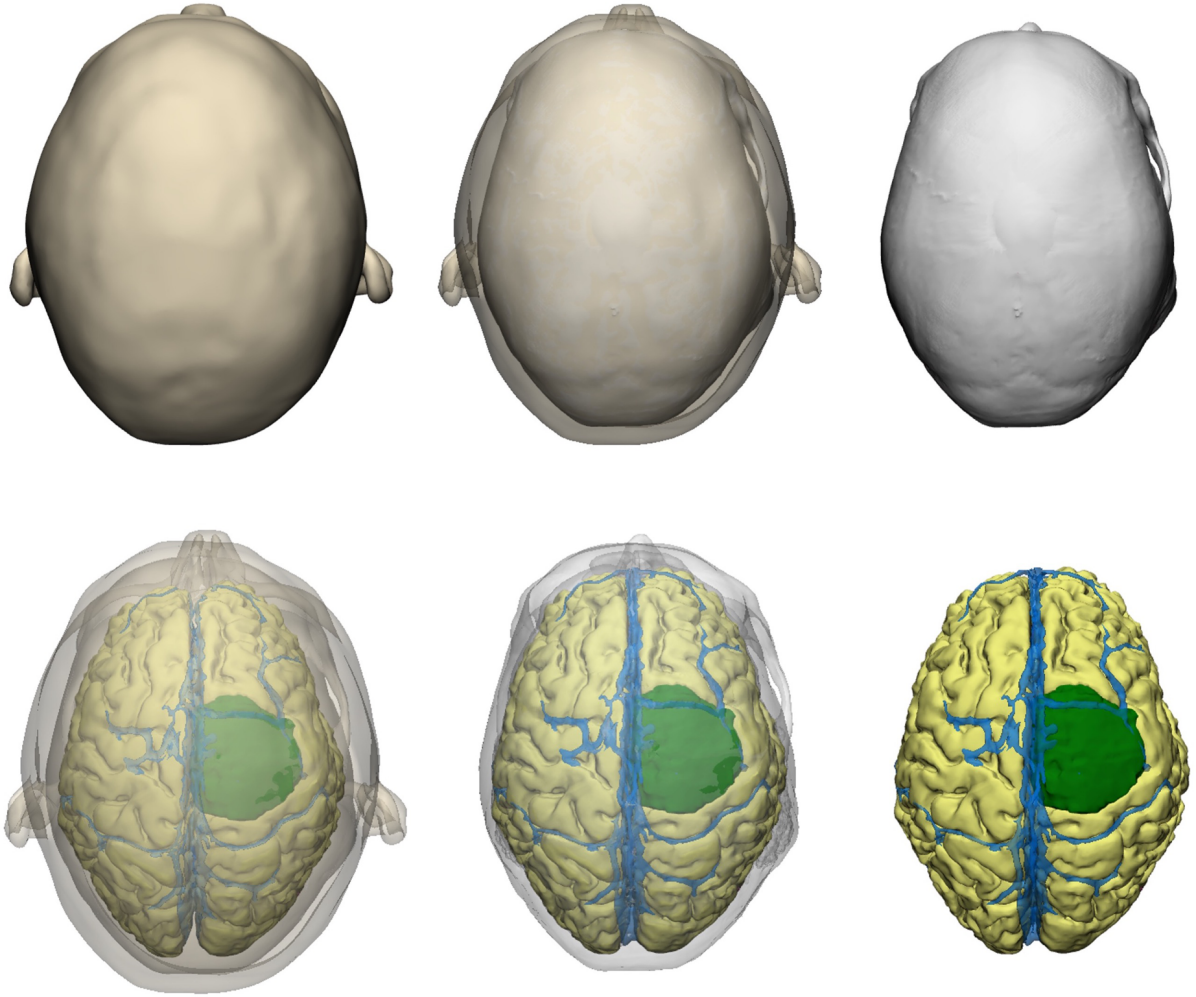
Visualization of various layers of the patient-specific 3D digital model.

For the preparation phase, one brain tumor case (40-year-old female with large parasagittal/parafalcine meningioma) was selected for this study. Neuroimaging data including volumetric MRI and CT scans were used for the reconstruction of the patient-specific digital 3D model. All 3D planning and modeling studies were carried out with Mimics Innovation Suite 22.0 Software (Materialise, Leuven, Belgium). Briefly, DICOM files of magnetic resonance imaging (MRI) or computed tomography (CT) scans were imported into Mimics. 2-dimensional radiological images were visualized on axial, coronal and sagittal planes. Masking process was undertaken using Hounsfield unit (HU) values on 2D radiological images. Segmentation of various structures was done according to anatomical borders. Different imaging sequences were used for segmentation of different intracranial structures: CT angiography for bone and vessels, T1W images with contrast for brain, ventricles and tumor (Figure 2). All CT and MRI scans were merged and aligned with the Align Global Registration module. Surface-rendering technique was used to produce 3D models of different anatomical structures. Then, a design module (3-matic 14.0, Materialise, Leuven, Belgium) was used for fine-tuning and detailed modeling. This allowed us to simulate and rehearse surgical scenarios by freely rotating, positioning, trimming, and adjusting transparency of the model and its components virtually (Figure 3).

### Planning and assessment session

**Figure 4:**
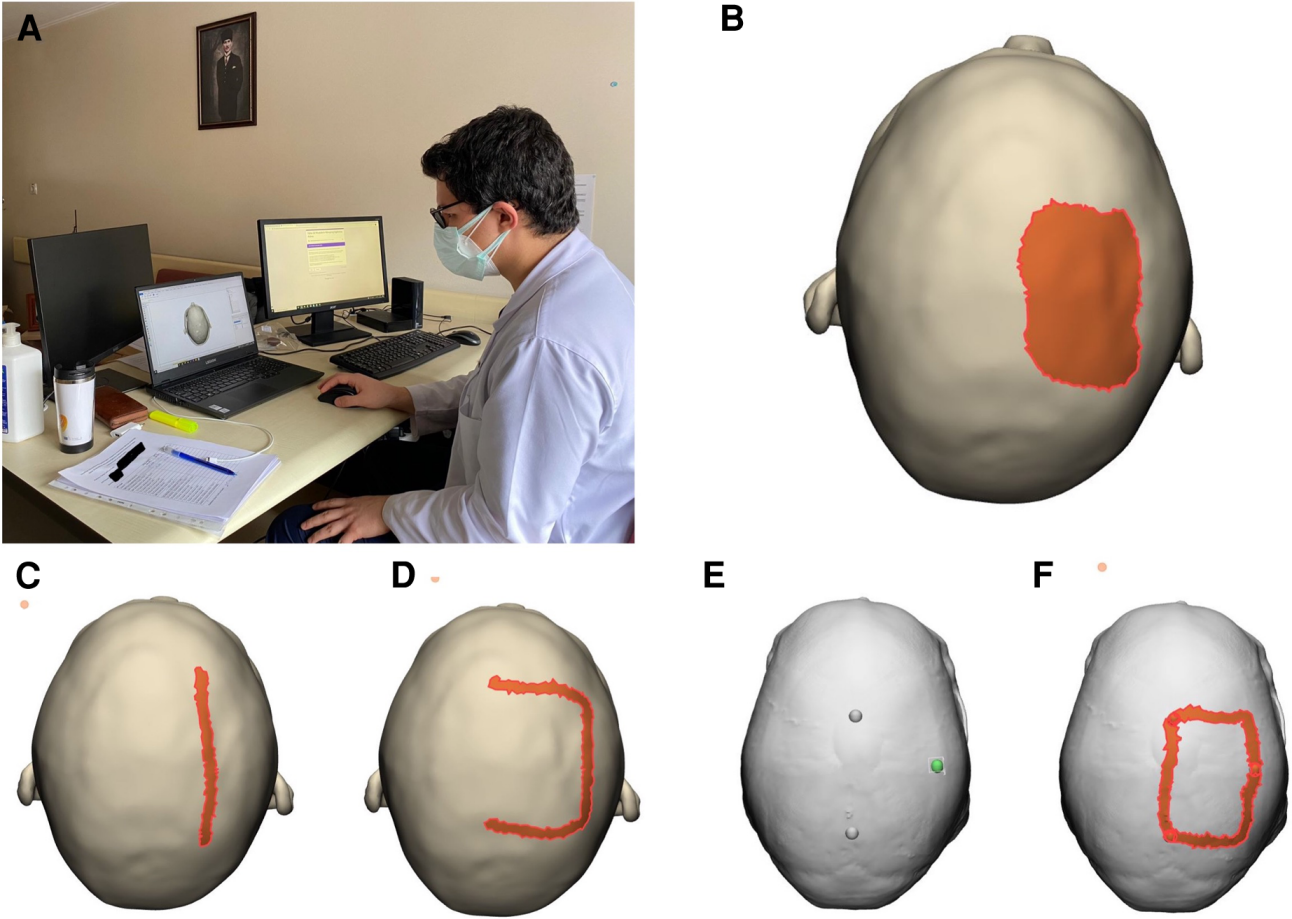
Planning and assessment session. (**A**) Photograph of the study setup. Drawings of (**B**) anticipated tumor projection on scalp, (**C**) linear incision, (**D**) U-shape incision, (**E**) burr holes and (**F**) craniotomy on two Accuracy Assessment layers (skin and bone) of the model.

A dedicated planning and assessment session in a quiet room was designed for this study. Two computers were used for the session, one for studying standard MRI as well as demographic data collection, assessment questions, and feedback, whereas the other was used for 3D model visualization and surgical planning assessment (Figure 4A). Participants followed the instructions for planning and assessment procedures with the guidance of an instructor who was not involved in the study design, assessment, and analysis (first blinding). After receiving a brief tutorial about the session and filling in the demographic and baseline information, the participant had 5 min to study the MRI examination (all 2D images at three planes) of the patient (planning with MRI). After that, they were asked to answer questions about the case (basic concepts, anatomy, radiology, surgical technique etc.). After these assessment questions, they were invited to perform virtual planning on the 3D model. For this accuracy assessment session, only opaque digital skin and bone models were used without exposing underlying tumor, brain, or intracranial vessels. Surgical planning steps included the drawing of (i) tumor projection on skin (painted area) (Figure 4B), (ii) linear and *U*-shaped incision (Figures 4C,D), (ii) burr holes and craniotomy borders (Figures 4E,F). Planning of each surgical step on the digital 3D model was individually recorded after each assessment session for each participant. After the Accuracy Assessment-1 (post-planning with MRI), the participant had a 5-min planning session studying the patient-specific 3D model. For planning with the 3D model, participants were free to use the 3D model to its full extent with all available viewer options (transparency, trimming etc.). After the second planning session, the participant underwent the same assessment again (questions, drawings) to better understand if there is an improvement in their surgical planning strategies. This assessment for the post-planning with the 3D model was regarded and recorded as the Accuracy Assessment-2. After all planning and assessment procedures were completed, participants were asked to provide their opinions (feedback) about the given statements by using a Likert-scale from 1 (strongly disagree) to 10 (strongly agree). The statements were about the realism, utility, practicality, and future potential of the digital 3D models (Table 1). All data were collected electronically using Google Forms. Surgical planning assessment data were saved as Mimics planning files.

**Table 1:** Feedback form and results.

Feedback (1: Strongly disagree, 10: Strongly agree)	Median [IQR]
Realism	
The digital 3D model shows anatomical structures realistically.	10 [9–10]
The digital 3D model realistically illustrates the superficial tumor and its relationship with surrounding structures.	10 [9–10]
The digital 3D model realistically simulates the surgical approach stages (skin incision, craniotomy, etc.).	9 [8.75–10]
The digital 3D model offers a realistic surgical planning environment for brain tumor surgery.	9.5 [9–10]
Performance	
The digital 3D model is good at showing skin and superficial landmarks.	8 [5–10]
The digital 3D model is good at showing bone and bony landmarks.	9 [7–10]
The digital 3D model is good at showing the surface anatomy (sulcus, gyrus).	9 [8–10]
The digital 3D model is successful in showing vascular structures (arteries, veins/sinuses).	9 [8.75–10]
The digital 3D model is successful in showing tumor boundaries and neighborhoods.	10 [8.75–10]
The digital 3D model is successful in showing fine anatomical details.	7.5 [4.75–9]
The digital 3D model is successful in reflecting the surface texture and color characteristics of anatomical structures.	9 [5.25–10]
Usefulness	
The digital 3D model is useful for understanding the relationship between different layers and structures in 3 dimensions.	9.5 [9–10]
The digital 3D model is useful for medical students’ anatomy education.	10 [9–10]
The digital 3D model is useful for neuroanatomy training of neurosurgery residents.	10 [10–10]
The digital 3D model is useful in determining patient and head position.	10 [8.5–10]
The digital 3D model is useful in choosing a patient-specific surgical approach.	10 [9.75–10]
The digital 3D model is useful for the development of surgical planning skills of neurosurgery residents.	10 [9–10]
The digital 3D model may reduce the possibility of errors in surgical planning.	10 [8–10]
The digital 3D model is superior to the cross-sectional MR study in terms of understanding the 3-dimensional relationship of the tumor with the surrounding structures.	8.5 [8–10]
Practicing with digital 3D models may improve surgical planning skills.	10 [9–10]
Practicing with digital 3D models may contribute to the improvement of surgical technique.	9 [7–10]
Practicing with digital 3D models may lead to faster acquisition of necessary skills.	10 [8.75–10]
Practicing with digital 3D models may help the surgeon during surgery by improving the ability to perceive individual 3D anatomy.	10 [9–10]
The inclusion of digital 3 models in surgical planning may increase the success of surgical procedures (extent of tumor resection, etc.)	10 [8.75–10]
Incorporating digital 3D models into surgical planning may reduce surgical planning errors and complications.	10 [9–10]
Practicality	
Working with a digital 3D model is confusing.	1 [1–2]
The digital 3D model is easy and practical to use.	9 [8–10]
Other opinions	
Use of digital 3D models in smartphone may contribute to its widespread use.	10 [7.5–10]
I would like to make more use of digital 3D models during my education.	10 [10–10]
The digital 3D model should be used in surgical planning prior to surgeries of complex and difficult cases.	10 [9.75–10]
The 3D digital model should be used in every cranial neurosurgery case.	6.5 [5–8.25]

### Assessment methodology

**Figure 5:**
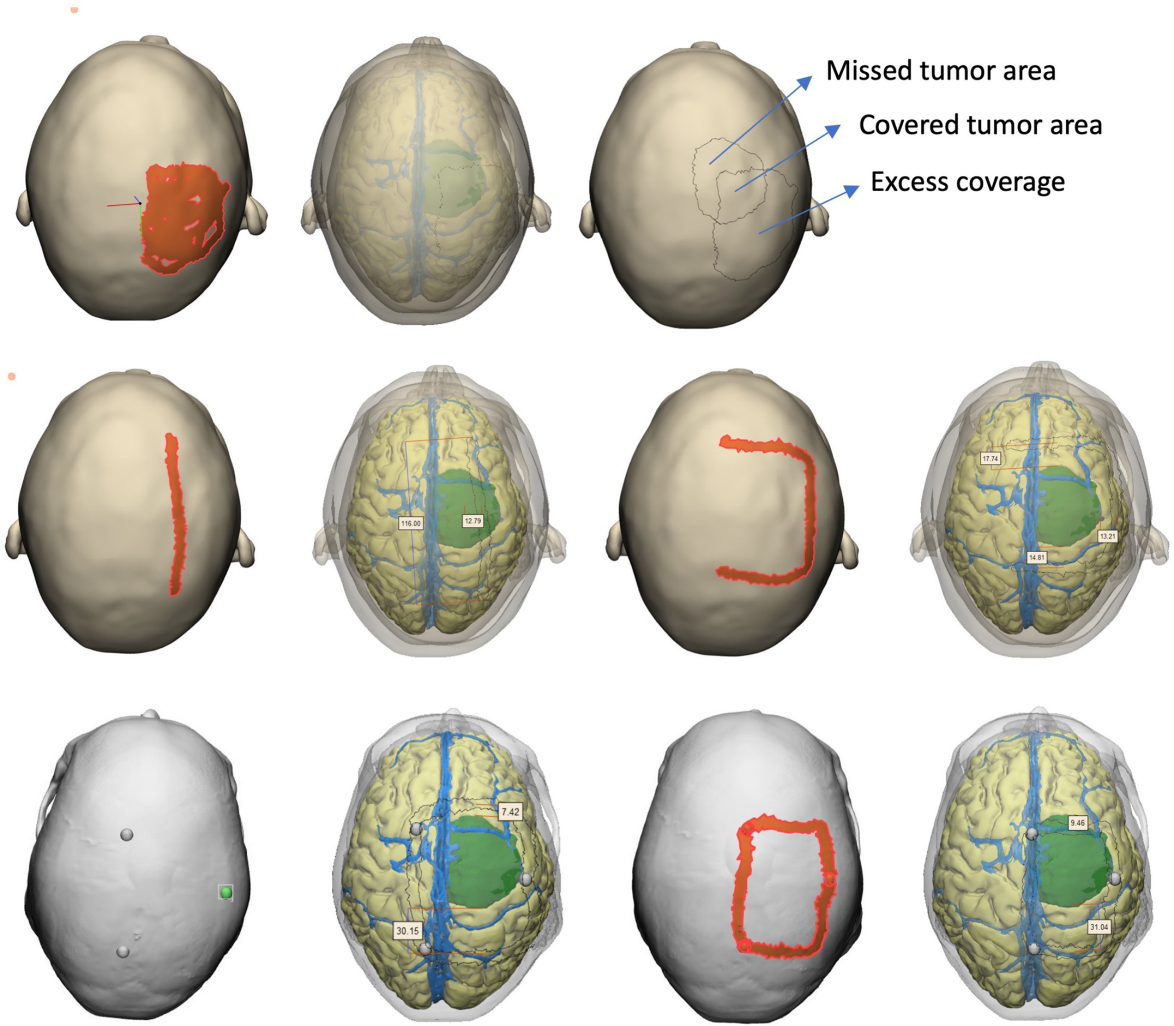
Assessment methodology. After saving each participant's drawings in digital format, assessors used the full model with transparency option to objectively measure the accuracy of drawings with reference to underlying tumor.

We aimed to assess the patient-specific radiological anatomy knowledge and surgical planning skills using the answers and drawings (i.e., surgical plans). The whole assessment was done by two neurosurgeons who were blind to identity and baseline information about the participant as well as the order of assessment (second blinding). The following parameters were measured and analyzed: (i) tumor projection on skin (percent tumor coverage, excess coverage), (ii) linear incision: deviation from center of tumor (mm), (iii) accuracy of *U*-incision (assessed by points from 0-the least accurate to 4-the most accurate), (iv) accuracy of craniotomy borders (assessed by points from 0-the least accurate to 4-the most accurate) (Figure 5). To calculate craniotomy score, each edge of craniotomy was scored 1 if it was within 5 mm–15 mm of tumor border (if three edges were within these limits, composite craniotomy score was 3). A similar approach was adopted for *U*-shape incision, the base of skin flap got an extra point if an imaginary line connecting two corners was within the limits. We also calculated the sum of deviation of 4 edges (margins) from the acceptable standard craniotomy borders (craniotomy margin deviation) as a more robust alternative to craniotomy score. For tumor projection, percent tumor coverage was calculated as covered (overlapping) tumor area/total calculated tumor area (2,717 mm^2^), whereas excess coverage was measured as the portion of painted area (as drawn by the participant) which did not match the underlying tumor (mm^2^).

### Statistical analysis

All statistical analyses were performed using the Prism 9 software (GraphPad Software, LLC., 2021). Descriptive statistics were presented using mean and standard deviation for parametric, median [interquartile range] for the non-parametric, count (percentage) for categorical variables. Paired *t*-test and Wilcoxon matched-pairs signed rank test were used to test whether there was a difference between measurements in Accuracy Assessment-1 (post-planning with MRI) and Accuracy Assessment-2 (post-planning with 3D model), for parametric and non-parametric variables, respectively. *P* value less than 0.05 was considered statistically significant.

## Results

### Baseline characteristics of participants

A total of 38 participants (14 neurosurgical residents, 24 interns) were included in this study. Mean age of residents was 29.7 ± 3.2 years, whereas it was 23.8 ± 1.2 for interns (6th year medical students). Four residents were in the first year (28.6%), two in the second year (14.3%), four in the third year (28.6%), three in the fourth year (21.4%), and two in the fifth year (14.3%) of residency program. Only two residents (14.3%) had prior experience with 3D modeling/3D printing/VR/AR.

### Surgical planning skills

**Figure 6:**
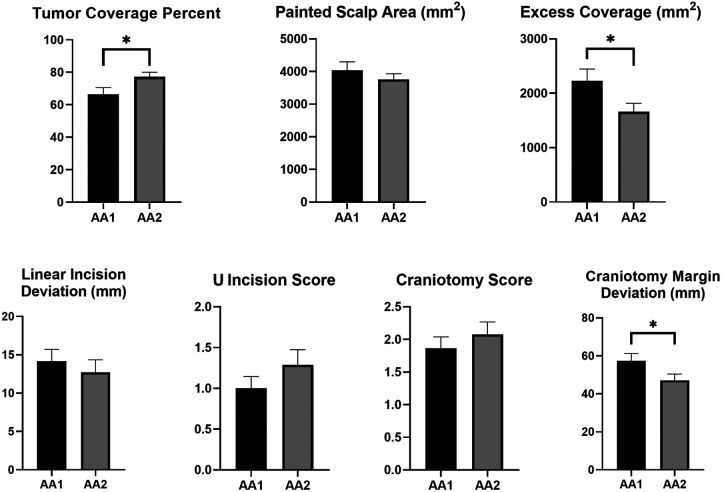
Change of the performance metrics between two assessment sessions. Accuracy Assessment-1 (AA-1): Post-MRI assessment, Accuracy Assessment-2 (AA-2): Post-3D model assessment. * represents significant difference at *p* < 0.05.

We compared the performance of participants on Accuracy Assessment-1 and Accuracy Assessment-2. For estimated tumor projection on scalp, percent tumor coverage increased (66.4 ± 26.2%–77.2 ± 17.4%, *p* = 0.026), and excess coverage decreased (2,232 ± 1,322 mm^2^–1,662 ± 956 mm^2^, *p* = 0.019) despite no significant change in painted area (4,036 ± 1,611 mm^2^–3,760 ± 1,081 mm^2^, *p* = 0.272) between Accuracy Assessment-1 and Accuracy Assessment-2 (Figure 6). For linear skin incision, deviation from tumor epicenter significantly reduced from 16.3 ± 9.6 mm to 8.3 ± 7.9 mm after training/planning with 3D model only in residents (*p* = 0.02) but not in interns. However, U-shape incision (1 [0–2] vs. 1 [0–2], *p* = 0.147) and craniotomy scores (2 [1–3] vs. 2 [1–3], *p* = 0.282) did not change significantly between two assessments. On the other hand, sum of craniotomy margin deviations from an acceptable standard decreased significantly after training with 3D model (57.3 ± 24.0 mm–47.2 ± 19.8 mm, *p* = 0.024) (Figure 6).

### Qualitative evaluation of digital 3d models

The participants scored realism, performance, usefulness, and practicality of the digital 3D models very highly (Table 1). The only aspect that they scored relatively lower was the demonstration of fine anatomical details [median 7.5 (4.75–9)]. Also, although the residents strongly agree that these models should be used for complex cases [median 10 (9.75–10)], they think that the 3D models are probably not necessary for every neurosurgical case [median 6.5 (5–8.25)].

## Discussion

Neurosurgical practice and education have seen tremendous change due to recent advances in medical, imaging and education technologies (1–3, 24). Coronavirus pandemic has further accelerated the digital transformation of our profession and the way it is thought and learnt (25). Hence, there is an everlasting need for more effective, realistic, and practical educational tools and methods to train the next generation of neurosurgeons (1–3). Despite the explosion of such tools and materials, objective validation is still lacking for many of them. In this proof-of-concept study, we validated the personalized-3D digital models as easily accessible, yet effective neurosurgical planning tools to improve the understanding of the 3D patient-specific anatomy and thus the accuracy of surgical planning for the first time in the literature.

First, we have showed that patient-specific digital 3D models are perceived as realistic, useful, and practical tools for education and surgical planning by neurosurgery residents (face validity). Second, we have demonstrated that their use as an assessment tool is also feasible, reliable and capable of generating numerous performance metrics for assessment of neurosurgical planning skills. Third, our study design allowed us to reveal that brief training with 3D model after a planning session with conventional 2D MRI slices provided an extra benefit in accurately predicting tumor projection on scalp and incision planning.

There are many studies evaluating the use of 3D digital and physical models as novel educational and objective assessment tools in the literature (14, 15, 19–21, 26). 3D printing has numerous advantages and applications, such as gaining better insight into patient-specific anatomy, better pre-operative planning, mock simulated surgeries, simulation-based training and education, development of surgical guides and other tools, patient-specific implants, bioprinted organs or structures, and counseling of patients (27). Numerous studies examined the efficacy of 3D-printed models on surgical planning, resident and patient education using quantitative and qualitative metrics (14, 15, 19–21, 26). Although it became widely accessible and used in recent years, patient-specific 3D-printing for intracranial pathologies are not routinely used in everyday practice largely due to their cost and production times. The incorporation of 3D printing technology in medical education and practice heralds a paradigm shift, offering unprecedented opportunities for innovation and advancement. Beyond traditional educational methodologies, 3D models empower learners to engage in hands-on experiences, fostering deeper understanding and retention of complex anatomical concepts. Moreover, the interactive nature of 3D models facilitates collaborative learning environments, where students and practitioners can explore anatomical variations and pathological conditions in real-time. As the technology continues to evolve, there is growing interest in harnessing artificial intelligence algorithms to automate the generation of patient-specific 3D models, streamlining the production process and enhancing accessibility. Despite the current challenges, the ongoing refinement of 3D printing techniques and the increasing affordability of equipment hold promise for the widespread adoption of patient-specific 3D printing in neurosurgical practice soon. It is inevitable that systems that support long learning curves, are patient-specific, and are used as standards in resident education, will be developed in the near future.

Virtual simulation-based training is also increasingly being used for assessment and training of psychomotor skills involved in many fields of medicine (28–31). A digital 3D model offers advantages over digital 2D or physical models in interactivity, perspective, access, and cost, either as a stand-alone learning asset or as part of a larger digital learning object (27). Recently, Ros et al. conducted a prospective randomized controlled study of 173 medical students to assess whether immersive virtual reality (VR) technique leads to improvement in learning the technique of external ventricular drainage. They showed that VR group demonstrated significantly better short-term results than the control group (*P* = 0.01). The same trend was seen at six months (32).

The application of artificial intelligence and machine learning technologies has also provided new methodologies to utilize large amounts of data for educational purposes. Mirchi et al. aimed to introduce a new framework using explainable artificial intelligence for simulation-based training in surgery and validate the framework by creating the Virtual Operative Assistant, an automated educational feedback platform with the contribution of twenty-eight skilled participants (14 staff neurosurgeons, 4 fellows, 10 PGY 4–6 residents) and 22 novice participants (10 PGY 1–3 residents, 12 medical students). Participants performed a virtual reality subpial brain tumor resection task on the NeuroVR simulator using a simulated ultrasonic aspirator and bipolar. The Virtual Operative Assistant successfully classified skilled and novice participants using 4 metrics with an accuracy, specificity, and sensitivity of 92, 82% and 100%, respectively (33).

Recent advances in imaging, image-processing and computer vision technologies provided various tools and software solutions to easily produce personalized or patient-specific 3D digital models with high resolution and quality (34–36). Their incorporation into daily neurosurgical practice provides vast opportunities for surgical planning, intraoperative guidance, and education. They can be combined or complemented with other technologies to enhance their potential as educational or surgical tools and simulators (37–40). Dho et al. recently established a 3D-printed brain tumor model production system, and their validation study showed significant superiority of the 3D-printed models in surgical planning regarding surgical posture (*p* = 0.0147) and craniotomy design (*p* = 0.0072) compared to conventional magnetic resonance images. They noticed that the benefit was more pronounced for neurosurgeons with less experience (14).

The prospects for application of digital 3D models, virtual reality and 3D printing in neurosurgical education are extensive (41). As the technologies advance, it is likely that current issues such as cost, and accuracy will be addressed and become less significant (20, 26). However, currently 3D printed models have certain limitations that prevent their frequent use. The requirement for specific printers and various materials, particularly for soft-tissue printing, makes the process expensive and lengthy. The success of 3D printed models also rely on the quality of digital 3D models created in a format recognized by a 3D printer. Rendering and segmentation of medical imaging data to produce patient-specific digital 3D models may also require special software and expert skills. Time and effort spent by an engineer, technician, radiologist, anatomist or biomedical illustrator to segment or create a digital 3D model add to the costs (42). However, with the advent of digital technologies and open-source software, these processes become more accessible, easier, faster and cheaper. We envision that the costs will diminish considerably in near future so that any neurosurgeon can use these patient-specific 3D digital models in everyday clinical practice. Therefore, there is a huge potential for their use in training, surgical planning, and educational assessment (43–45). This potential should be further explored by neurosurgeons, educators, trainees and professional organizations.

One of the main challenges in assessing the utility of virtual educational tools is the need of an objective evaluation method for performance or learning (46, 47). Most virtual simulators designed for neurosurgical education depend solely on the appraisal of resident/trainees' subjective judgements and lack the objective, measurable and repeatable parameters or metrics necessary to validate the usefulness of the system (33, 47). Presented is the first study in the literature to assess the effects of incorporating a patient-specific 3D virtual model for educational purposes, on incision and craniotomy planning skills of neurosurgery residents. Our study design enabled us to monitor the improvements in accuracy of linear incisions and corresponding craniotomies made by residents in a blinded fashion. They were asked to draw perceived tumor projection, a linear and a horseshoe (U-shaped) incision on scalp layer of the 3D model, and corresponding craniotomy on bone layer of the 3D model. Evaluation methodology was simple, yet objective, quantitative, measurable and reproducible. The literature consists of both more complex simulation scenarios such as skull base tumors or aneurysms clipping and simpler ones such as external ventricular drain placement. The assessment methodology used in our study also showed that numerical parameters such as distance, area, percent area provide more powerful and accurate metrics than rough scoring measures such as those used in the U-incision or craniotomy scores in the current study. Also, the study design allowed a fair comparison of the use of 3D model with conventional neurosurgical planning (i.e., examining 2D MRI sections). We did not elect to compare two techniques, rather evaluated the utility of 3D model as a complementary tool as in clinical practice. Indeed, brief training with 3D model after conventional planning led to significant improvements in performance metrics.

Despite its several strengths, the current study has also some limitations. First of all, this is proof-of-concept study and therefore focused on only one surgical case and a handful of performance metrics. It's important to note that the evaluation of performance metrics in this study was constrained by the necessity to manage session duration effectively. Since session time is important for such performance assessment studies, we had to limit our study to only one case and a few metrics as the session duration was already over 40 min (tutorial, collection of demographic data, practice with MRI scan, first assessment, short break, practice with 3D model, second assessment, feedback) even for a single case. Second, we had a relatively small number of neurosurgical residents and interns from one institution. Third, we used the same digital model, but with only skin and bone layers, for assessment. This may have artificially increased the chance of improvement with the use of 3D model as a planning tool over conventional planning with MRI. Alternatively, we may have used real surgical scenario in the OR, or a physical anatomical head model. Nevertheless, we believe that using the same model also enabled us to perform more precise and objective measurements specific to the individual pathological anatomy. Also, its fully digital nature enables repetitive use, multiple recordings, and retrieval of recorded planning data. Future studies with larger cohorts including experts and trainees at different levels, and with variety of surgical scenarios (e.g., deep brain tumors, skull base and vascular pathologies) and performance metrics (precision, depth perception, time, number of errors, appreciation of 3D relationships etc.) can further explore the potential of personalized digital 3D models (i.e., content, construct and criterion validity) and establish the optimal methodology to use their full potential in neurosurgical training and practice.

## Conclusion

Digital 3D images, models, and simulators are useful for developing surgical planning skill set. However, their standardization and validation are equally important for their incorporation into neurosurgical curriculum. Here, we showed that readily accessible patient-specific digital 3D models can be effectively used both as educational and assessment tools by evaluating a trainee's perception of intracranial lesions through their imagination of tumor projection on scalp and decisions such as skin incision and craniotomy placement. This tool has many advantages including no or minimal cost, numerous virtual simulation options, repetitive use, archiving possibility, objective measurements, suitability for remote or distant assessment and analysis. They can be incorporated into standard surgical planning workflow of residents, and also be used as a skills assessment tool in local programs as well as national or international board examinations. Therefore, neurosurgery education can benefit from these tools and methodological approach described in this study.

## Data Availability

The raw data supporting the conclusions of this article will be made available by the authors, without undue reservation.
